# Language and “Theory of Mind” development of bilingual and monolingual children in Bulgaria

**DOI:** 10.3389/fnhum.2025.1522507

**Published:** 2025-10-20

**Authors:** Huseyin S. Kyuchuk

**Affiliations:** Faculty of Arts and Educational Science, University of Silesia in Katowice, Katowice, Poland

**Keywords:** preschool children, bilingualism, Turkish, Bulgarian, Theory of Mind, wh-complements, evidentiality, L1 and L2

## Abstract

Children from Bulgaria (*N* = 120) were tested on language and Theory of Mind (ToM) development. Sixty were ethnic bilingual Turkish children, and 60 were monolingual ethnic Bulgarian children. The age of the children varied between 3;6 to 5;0 years old. Both groups of children in the study were tested in their mother tongues (Turkish and Bulgarian); the Turkish children were also tested in their second language (L2)—Bulgarian, with a language test and Theory of Mind test. Theory of Mind was tested with classical tasks plus a non-verbal ToM task, and the language test comprised measures of wh-complements and evidentiality marking. The hypotheses tested were: H1: The comprehension and production of wh-complements in the mother tongue (L1) at ages 3–6 years will support the understanding of ToM in their second language (L2). H2: Understanding “evidentiality” marking in the mother tongue will support an understanding of false belief ToM tasks in both languages. The results show that the Turkish-speaking children had a lower level of understanding of the classical ToM tasks than the Bulgarian monolingual children, but have equivalent results on the non-verbal ToM task. In the language test, the Turkish-speaking children were better in wh-complements, but weaker in performing the evidentiality test than the Bulgarian monolinguals. The predictors of performance in classic ToM tasks were different from the two ethnic groups: for the Bulgarian monolinguals, performance on the evidentiality test was the best predictor, but for Turkish bilingual children, performance on the low verbal tasks was the only predictor other than age, for both L1 and L2 ToM.

## 1 Introduction

According to Schroeder (2018, 2), “Theory of Mind refers to the ability to attribute mental states to other people and to predict and explain other people’s behavior on the basis of those attributed mental states. This ability is often assessed through a false belief test, such as the unexpected-transfer test and the unexpected-content test.” To solve these tests, children must recognize that a character has a belief counter to a reality that the child knows.

Wellman et al. (2001) raised an important question about the universality of theory of mind development in children across different languages and cultures. Their metareview strongly suggests that there are strong similarities across languages and cultures in the patterns of growth of the child’s abilities to take into account the desires, beliefs and knowledge of other people. Yet at the same time, several decades of work have shown significant environmental influences on the variability in children’s success or rate of development, such as the number of siblings in the home (Perner et al., 1994), and the amount of language about the mind that the child hears (Dunn, 1996; Ruffman et al., 2002; Koç et al., 2024). Furthermore, the contribution of the child’s own language development is strongly implicated in a number of publications (Ruffman et al., 2002; Astington and Baird, 2005; Farrar et al., 2017; de Villiers and de Villiers, 2025; Boeg Thomsen et al., 2021; Brandt et al., 2023), especially vocabulary, general syntax, and particular skills to do with the vocabulary and syntax needed to express mental states such as belief. Despite the increasing numbers of publications, the range of languages and cultures explored is still quite narrow, and the contribution of linguistic properties such as evidential marking, found in many languages, is still unclear (Aksu-Koç, 2005; de Villiers and Garfield, 2017).

The present study is the first one with Turkish-Bulgarian bilingual children focusing on the development of Theory of Mind. Both languages have the two grammatical phenomena-sentential complements and evidentiality – that have been previously implicated in ToM, allowing an exploration of the role they might play in the children’s ToM development, compared to Bulgarian monolinguals.

Bilingual children’s ToM development has come in for special scrutiny in the last two decades. An important question concerns how children who are learning multiple languages perform on Theory of Mind tasks. Goetz’s (2003) classical study on ToM of bilingual children tested three- and four-year-old English monolinguals, Mandarin Chinese monolinguals, and Mandarin-English bilinguals. The children received appearance-reality, level 2 perspective-taking, and false-belief tasks. The monolinguals were tested in their mother tongues and the bilinguals in each of their languages. The results showed that the 4-year-olds were better than the 3-year-olds, but also that the bilinguals performed significantly better than the monolingual groups. Nguyen and Astington (2014) also investigated whether the bilingual advantage in false-belief (FB) understanding was replicated when socio-economic status was considered. Again, results showed that bilinguals significantly outperformed monolinguals on FB tasks. Similarly, Huang et al. (2023) focused on children in poverty and investigated whether early bilingualism enhances economically disadvantaged children’s Theory of Mind (ToM). Results again showed that bilingual children demonstrated greater ToM competence compared to monolingual children. In a meta-analysis of 16 studies, Schroeder (2018) found that bilinguals are advantaged over monolinguals. Feng et al. (2023) analyzed 53 publications examining ToM of bilingual children, and most of the papers showed that bilinguals show higher results in the performance of Theory of Mind tasks than monolinguals.

What are some theories of bilingual advantage? Buac and Kaushanskaya (2019) examined whether language and Executive Function (EF) skills were predictive of Theory of Mind (ToM) performance in bilingual and monolingual children. The authors found that language ability predicted ToM performance in simultaneous bilinguals but not EF skills. Schroeder (2018) also suggested that bilinguals have superior metalinguistic awareness and socio-pragmatic abilities that promote ToM. Diaz and Farrar (2018) found that metalinguistic skills predicted ToM for their bilingual subjects.

Perhaps more data is needed on a wider range of children, circumstances and languages before the answers will be clear for the explanation of the bilingual advantage in ToM. The range of languages studied with bilingual participants is still restricted, as are the sociolinguistic and economic conditions of the children in the studies. The motivation for the current study was to find out how the Bulgarian Turkish minority children perform ToM tasks. These two languages belong to very different language families, and they have been neglected in previous work. The major question was how the language skills being measured might help the children to understand Theory of Mind.

Being bilinguals, the Turkish-speaking children, as Kroll and McClain (2013, 112): “can choose the language they wish to speak, but at the same time switch back and forth from one language to the other, often in midsentence, with others who are similarly bilingual. The evidence suggests that the consequences of bilingualism are largely positive, with features of bilingual minds and brains reflecting the benefits of a life spent negotiating the presence of two languages and acquiring the skills to select the appropriate language in the intended context.” It suggests that learning the syntax in the first language could open the door for ToM understanding in the second language, especially if the tasks were nonverbal. Usually, the bilingual speakers are sensitive to cultural codes of the languages they speak and in conversations they follow these rules. All these codes are acquired from a very early age.

In a number of studies and publications over the last approximately 20 years, Kyuchukov (2000, 2002, 2007, 2009, 2018, 2019) and Kyuchukov and Giray (2017) have sought to show how bilingual Turkish-speaking children from Bulgaria and Germany learn their mother tongue and the official languages of the countries where they live—Bulgarian and German. The authors’ research shed new light on different aspects of language development—morphology, syntax, pragmatics and the importance of both languages for their school success. A study with Turkish-Swedish and German-Swedish bilingual children in Sweden (Bohnacker et al., 2016) determined from empirical data that children whose parents used the home language with their child had significantly higher vocabulary production scores. Kabadayi (2008) analyses the second language acquisition of Turkish-speaking bilingual children living in Germany and the author observed the socio-demographic structure of the Turkish bilingual children, of their parents, and children’s opinions about second language acquisition. The author found that the socio-economic status of the family and the socio-demographic structure influences the process of language learning.

It is sometimes found that bilingual children from families with low socio-economic status have difficulties in acquiring a second language, because their L1 is not very well developed either (Xu and Liu, 2021). Akoğlu and Yağmur (2016) compared the language knowledge of bilingual Turkish-speaking immigrant children in the Netherlands and monolingual Turkish children in Turkey. The authors found that compared to monolingual Turkish speakers, Turkish immigrant children were behind in their first language in both cognitive concepts, and lexical, syntactic, and textual skills. The authors claim that the education of the mother plays an important role in the performance of the tasks of immigrant children. It has an effect on their cognitive development as well as on their second-language acquisition. A study with Turkish children in Belgium reports that the mother tongue of bilingual Turkish students is considered to be a barrier to educational and occupational success. The bilingualism of Turkish-speaking children in the case of Belgium is not seen as an asset (Agirdag, 2010).

In contrast, Akinci (2001, 2008) examined language and perspective-taking across both languages of Turkish-French bilinguals exposed to Turkish educational programs. Akinci found that the children ended up with Turkish language skills superior to those of their parents who were raised and grew up in Turkey, but may have had more limited access to education.

As can be seen, the language situation of bilingual Turkish-speaking children is very diverse. In most of the cases there is language assimilation towards the official language of the country where the Turkish children live. However, both languages (L1 and L2) of bilingual Turkish-speaking children may be important for the development of their Theory of Mind.

Most of the studies on Theory of Mind with Turkish-speaking children have been carried out with monolingual children. In a study with monolingual preschool Turkish children, Aksu-Koç (2005) found that the Turkish-speaking children achieve earlier false-belief understanding as compared to English-speaking children reported in the literature. The authors also found that control of *evidentiality* and of *complement constructions* were significant predictors of false-belief performance and suggest that these two factors are also important contributors to ToM development.

In other work, Ozoran (2009) studied monolingual Turkish children between 4 and 7 years old, testing with both an evidentiality task and comprehension of relative clauses, found that competence in relative clauses was a significant predictor of ToM performance. The past tense marker -DI was clearly understood by the children, but not the evidentiality marker-MIS. The author concludes that the -MIS marker does not develop until 6 years old and this is why evidentiality cannot be a predictor of Theory of Mind development.

Bodur (2022) investigated the use of complement clauses by mothers in relation to comprehension of complement clauses by children and Theory of Mind development among 3- to 5-year-old Turkish-speaking children. The children were given comprehension of complement clauses task, expressive and receptive language tasks, and three ToM tasks. The results showed that the frequency of complement clause structures in the mothers’ speech was however, not significantly related to children’s comprehension of complement clauses. The frequency of complement clauses that include mental state verbs was not significantly related to ToM. According to Csató (2009) double complement clauses in Turkish occur when one complement clause is embedded within another, formed by using nominalization suffixes like -mEk or -mA on the embedded verb and then applying another nominalization or a case suffix. Children’s comprehension of double complement clauses was significantly related to ToM, but the correlation was not significant after controlling for the children’s expressive and receptive language.

Selcuk et al. (2018) found that large households help for earlier understanding of varying belief states as well. By reviewing the literature on the age and pattern of ToM acquisition in Turkish-speaking children as well as the social and cognitive factors linked with Turkish children’s ToM development, Ekerim-Akbulut (2022) highlights both culture-specific, SES and universal patterns in mental state understanding.

There is just one study on ToM of bilingual Turkish-speaking children in Germany (Kyuchukov, 2023). That study, conducted with two groups of bilingual ethnic Turkish children between 4 and 6 years old, aimed to examine the influence of the mother tongue (L1) and the second language (L2) on the understanding of the Theory of Mind. The children were administered the classical tests for ToM, as well as language tests related to the comprehension of wh-complement sentences, containing a mental state verb and comprehension and production of vocabulary in native Turkish and German as a second language for them. The results showed that vocabulary was not an important factor, and the mastery of wh-complement sentences was the factor that enabled children to understand the false beliefs.

The literature about language and Theory of Mind in Turkish-speaking children clearly has mixed results. The aim of the present empirical study is to determine to what extent the proficiency of different grammatical categories in the mother tongue L1 and in the second language L2 of Turkish bilingual children affects their understanding of Theory of Mind. Accordingly, a central question addressed in this study is:

*How does the development of wh-complements and evidentiality marking connect to children’s ToM development, in bilingual Turkish children with Bulgarian as L2?*

The objectives of the study are to ask:

1. How wh-complements and evidentiality as grammatical features of Turkish assist Turkish-speaking bilingual children to understand verbal and non-verbal “False-Belief Tasks” (FBT).
2. How Turkish-speaking bilingual children perform Theory of Mind and each of the language tasks in each of their two languages: Turkish as first language (L1) and Bulgarian as a second language (L2), and how the results relate to other bilingual studies described in the literature.

## 2 Turkish community in Bulgaria

Bulgaria has a large Turkish minority. Many Turkish families from Bulgaria have relatives in Turkey, and they travel very often to the neighboring country of Turkey. There are Turkish satellite TV channels which can be readily watched in Bulgaria. Before 1990, during the communist regime in Bulgaria, the connections between the two countries were very poor. The Turkish minority was oppressed, the names of all Muslims were changed and the use of Turkish and any other minority languages in public places was forbidden. The minority children did not have the right to speak their mother tongues in kindergartens and schools and there were no lessons in Turkish as a minority language (Rudin and Eminov, 1990; Marushiakova and Popov, 2004; Kyuchukov, 2019).

After the democratic changes in 1990, the government allowed the minority children to have L1 literacy instruction in their mother tongue together with the official language of the country—Bulgarian. The children now study Turkish in 4 lessons per week (Kyuchukov et al., 2024). Almost all Turkish children speak the local variety of Turkish at home (Northeast variety of Turkish), because they live in a region with a predominantly Turkish community. At school they learn Bulgarian, but their competencies in Bulgarian are initially limited (Kyuchukov, 2019). The Turkish children learn their mother tongue from the extended families. Most of the Turks live in villages and small towns, where the family connections are intense. The children have contacts with family members as well as with community members where they learn the codes of the communication. From an early age, the children participate in community life and in all kinds of celebrations and holidays, and this is how they learn the grammatical rules of conversational Turkish.

The Bulgarian children grow up in a nuclear family and they learn Bulgarian from communication with the mother, father and siblings. The language is presented to the children through book-reading, toys, listening to fairy tales and songs, often now from tablets or phones.

What is unique about the linguistic properties of Bulgarian and Turkish that might be connected to Theory of Mind? There are two grammatical differences to highlight. One concerns the nature of wh-question complements, which have been implicated in theories of how language assists false beliefs reasoning. The second is evidentiality (Papafragou and Li, 2001; Aksu-Koç, 2000), namely, the grammatical marking of source of knowledge on verbs. Evidentiality has been linked to Theory of Mind because it indicates how the speaker comes to know the truth of what they are saying-whether it is from hearsay, direct witnessing, inference and so on. Both Turkish and Bulgarian have grammatical marking of evidentiality.

### 2.1 The wh-complements in Balkan Turkish and Bulgarian

In standard Turkish, the wh-question is in-situ, that is, wh-words do not exhibit obligatory movement to sentence-initial position in wh-questions like they do in English. Wh-phrases in Turkish do not have to be placed at the beginning of a sentence (Göksel and Kerslake, 2010: 211):


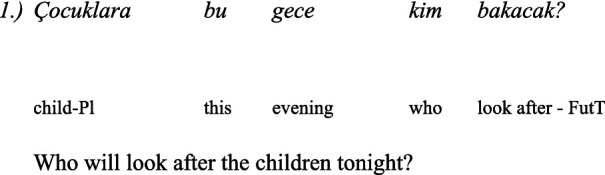


However, in Balkan Turkish, according to Kuteva and Heine (2012) the wh-words could be placed at the beginning:


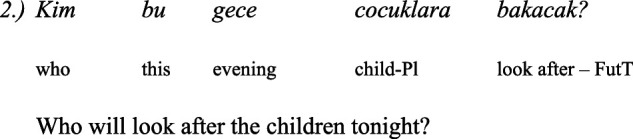


In Bulgarian, the wh-question is in the fronted position as in English. For example:


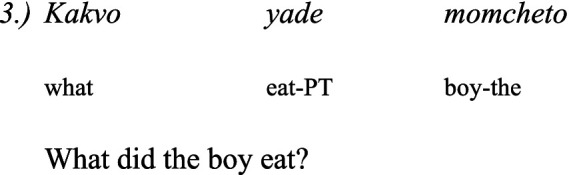


Comparing the wh-sentences in Bulgarian and in Balkan Turkish it seems the Balkan Turkish wh-complements are influenced by Bulgarian (Kyuchukov, 2024).

### 2.2 Evidentiality in Turkish and Bulgarian

There is some controversy among linguists about the correct analysis of the morpheme -*mIş* and -*DI* in Turkish, and whether they are truly evidentials. Gül (2009, 177) reports that “Evidentiality in Turkish is coded by the verbal suffix *-(I)mIş*.” The author discusses the role of the morpheme *-mIş* in Turkish grammar, since the morpheme plays the role of past tense suffix, but it can be called the “hearsay past marker.” In other accounts, *-mIş* is the called the perfective marker of Turkish. Some linguists specifically refer to the *-mIş* morpheme as marking indirect evidence, that is, inference and hearsay. The marker *-DI* used as past tense is used when something has been witnessed directly.

Under the Turkish influence, Bulgarian developed evidentiality marking as well. In linguistic literature this issue has been well investigated. According to Sauerland and Schenner (2019), Bulgarian has at least 3 kinds of evidential markers:

1. Direct(dir) (aka confirmative, witnessed)
2. Reportative(rep) (aka nonconfirmative, renarrative, perfect of evidentiality)
3. Dubitative(dub)

Aikhenvald (2004) has a review of evidentiality types across languages, and classifies Bulgarian as either type A1: *Firsthand* vs. *Non*-*firsthand,* or as type A2: *Nonfirsthand* vs. *everything else*. In Bulgarian, the Direct evidential is null, that is, there is no specific morphological marker.


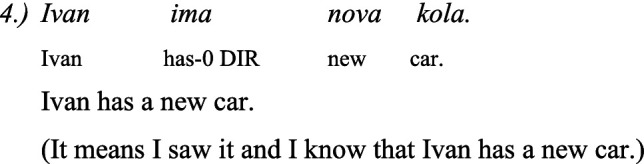


The Reportative evidentiality has the same marker as the perfect form:


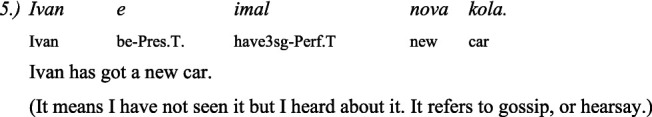


The dubitative evidential is similar to the reportative and it is morphologically realized in a periphrastic form be+perf and participle+perf, as it is shown in example 3.


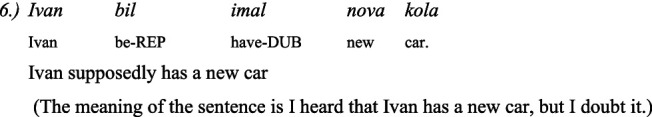


The Turkish evidentiality is expressed with the morpheme *-mIş* and *-DI* on the end of the verb and the evidentiality in Bulgarian is expressed with the morpheme *-l, −la, −lo, −li* in the end of the verb.

Generally speaking, the evidentiality in both languages has the role of passing information about the source of knowledge. Hence, it has been connected to Theory of Mind. The understanding of the meaning of evidentiality is also connected with language development and intertwined with acquisition of grammatical categories such as tense and aspect by young speakers.

## 3 Methodology of the study

Research hypotheses of the study are:

*H1*: Comprehension and production of wh-complements in the mother tongue (L1) at ages 3–5 years will support the understanding of false beliefs in the mother tongue in both ethnic groups.

*H2*: Comprehension of grammatical category of “evidentiality” in the mother tongue will support an understanding of false-beliefs in both groups.

### 3.1 Overview of the methodological approach

In order to achieve the aims and objectives of the study and to substantiate or reject the hypotheses put forward, a comparative study was conducted with ethnic Turkish bilingual preschool children (between 3 and 5 years) and ethnic monolingual Bulgarians of the same age, both from Bulgaria.

The empirical study was conducted with Turkish-speaking children from a small town in northeastern Bulgaria, where one segment of the Turkish population is concentrated. The ethnic Bulgarian children are also from northeastern part of Bulgaria and from the same town. All children attend a kindergarten, where the educational process is organised in Bulgarian language.

### 3.2 Participants

The total number of children involved in the study is presented in Table 1.

**Table 1:** Participants information.

Groups	1^st^ gr.: 3; 6–4; 0 years old	2^nd^ gr.: 4; 1–4; 6 years old	3^rd^ gr.: 4; 7–5; 0 years old	Total
Experimental group: Turkish	20	20	20	60
Control group: Bulgarians	20	20	20	60
Total	40	40	40	120

A total of 60 bilingual Turkish-speaking and 60 monolingual Bulgarian-speaking children were involved in the study, amounting to 120 children in total.

### 3.3 Research methods

The bilingual children included in the study attend kindergarten at around the age of 3;0–3;6 years old and this is their first contact with Bulgarian language. Until this age they speak at home mainly Turkish which is their mother tongue and later they start learning Bulgarian. The experiment was conducted in both languages in the kindergarten environment. The ethnic Bulgarian children are tested only once in their mother tongue of Bulgarian.

### 3.4 Tests

Two types of test were employed: those that measure understanding of ToM, and language tests. The tests are described as follows:

#### 3.4.1 Tests of understanding of the False-Belief Tasks (FBT)

Which are well known in psychology and have become classic in studying ToM. They are known in the English-language literature as the *Unexpected contents task (Smarties)* and the *Change of location task (Maxi).* In a range of different psychological studies, they have been used in different cultural contexts, with different objects and illustrations, but with the same content.

In *the Unexpected content* (*Smarties*) task, the child is shown a box/package of something with the contents not visible and asked the question: *What do you think is in the box/package?* Usually the child answers what is illustrated in an image on the package (candy, cookies). The box is opened and the contents are found to be something else—a toy, a pencil. The box is closed and again the question is asked: *What did you think was in the box when you first saw it? And what was in the box?*

The second task involves *changing the location* of an object (ball). It was adapted for the purpose of the study from the *Sally-Anne Theory of Mind test* by Baron-Cohen et al. (1985). First two protagonists (two toy animals) placed the ball in one location, then one protagonist leaves the scene. In the absence of the first protagonist, the second protagonist changes the location of the ball. The first protagonist returns to the place of action and the child is asked: *Where will he/she look for the object?*

The two tasks have been modified and adapted for the purpose of the study in Bulgaria, testing Turkish and Bulgarian children respectively, but the content of the tasks has not changed. The materials, boxes, puppets used for Turkish and Bulgarian testing were changed so the children do not know the answer on the second testing. These two tasks are verbal. Along with the two verbal tasks, the children were administered two low-verbal FBTs. They are presented to the children with pictures and drawings. These were used to minimize the language demands of the tasks on the children.

The low verbal task required the child to look at a pair of pictures, for example shown in the picture, and again, to fill in the thought balloon about what the person thinks is under a flap. These methods have been used successfully with deaf children in previous research on false belief reasoning.^1^ There were 4 such examples.

#### 3.4.2 Language tests

Children were given two language tests. The first test contained comprehension of *wh-complement questions* with a communication verb. In the study this test had 5 items. The language tests were in local dialect of Turkish spoken by the children and in official Bulgarian.

In local Turkish:


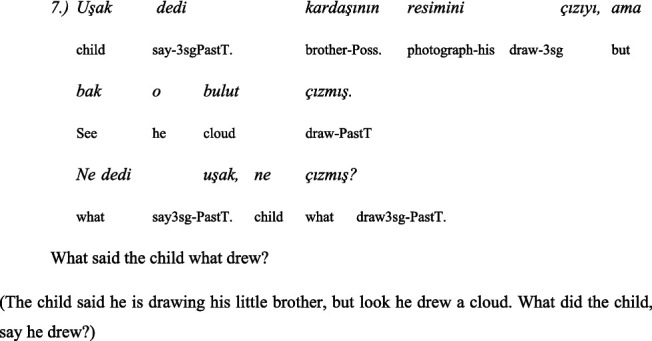


In Bulgarian:


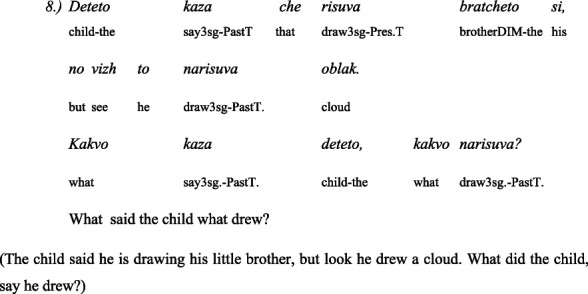


The second linguistic task was to understand the grammatical category of *evidentiality* in both Turkish and Bulgarian. The children administer 5 pairs of items: the same text in two versions—the first version is in the *past perfect tense* and the second is with the indirect *evidential marker*. The texts are narrated by two toy animals, a dog and a cat. One time the dog narrates the text in the past perfect tense and the cat with the indirect evidential, and the next time the roles are reversed: the cat narrates in the past perfect tense and the dog in the indirect evidential. This avoids associating the dog with past tense and the cat with indirect evidential.

Here are some examples for a pair of past tense and indirect evidential phrases used in the test of evidentiality (Kyuchukov and de Villiers, 2009):

#### 3.4.3 In Bulgarian

##### 3.4.3.1 Story: the dog tells it (in past tense)


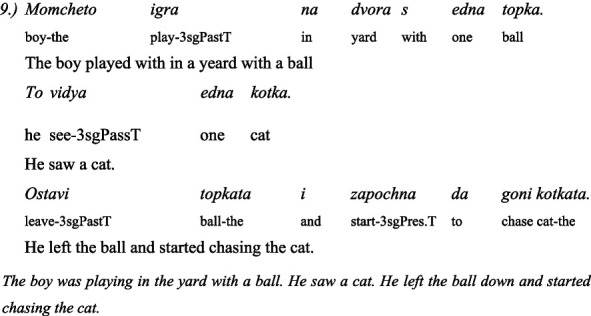


##### 3.4.3.2 Story: the cat tells (with indirect evidentials)


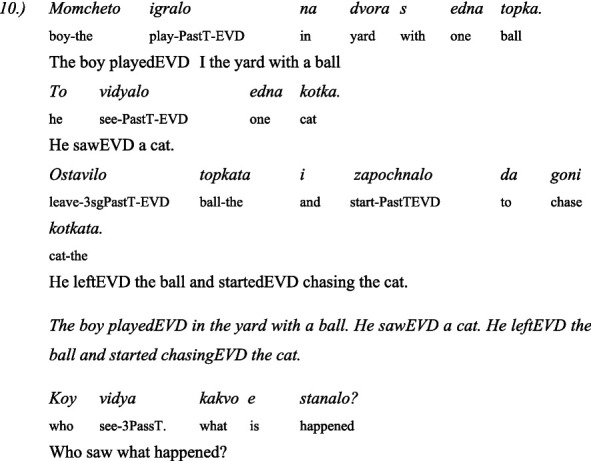


#### 3.4.4 In Turkish

##### 3.4.4.1 Story: the cat tells (in past tense)


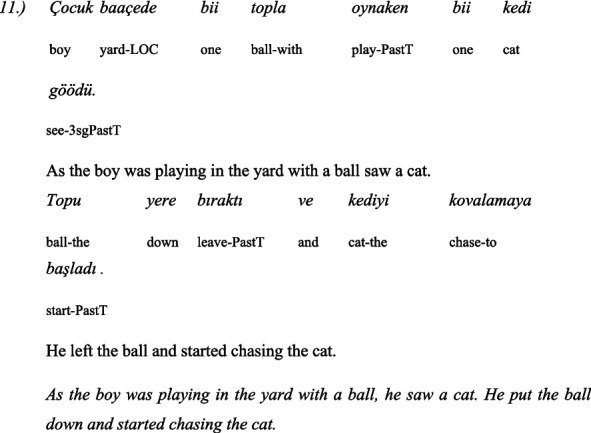


##### 3.4.4.2 Story: the dog tells (with indirect evidentials)


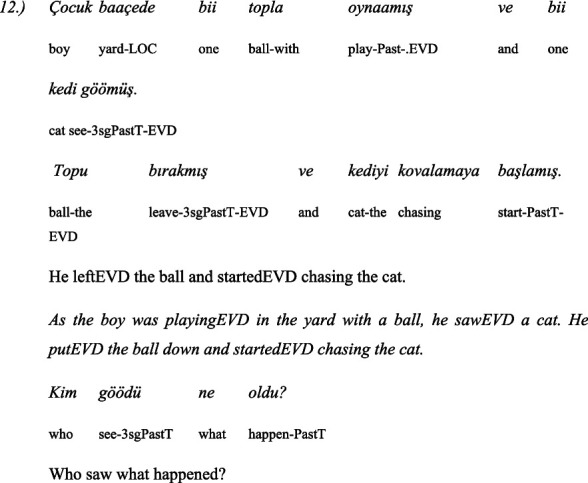


### 3.5 Ethics and procedure

The research study design and protocols were submitted to the University Ethic Commission and Regional office of the Ministry of Education in Bulgaria for their review and were approved. The content of the tests was not considered a risk for the psychological health of the children.

Each child was tested with the tests described above. Mother tongue testing was carried out by the researcher who is a native speaker of the Turkish dialect spoken by the children. Testing in Bulgarian language was conducted by a teacher working in the kindergarten. The testing was carried out in the kindergarten in a separate room individually with each child.

## 4 Results

The data was analysed with the statistical package SPSS. First, I present the results of both ethnic groups children in their mother tongues—Turkish and Bulgaria. Then the results of Turkish children in Bulgarian as a second language and Bulgarian of Bulgarian children as a mother tongue will be presented. Finally, the results of the Turkish children in their mother tongue of Turkish and in their second language of Bulgarian will be presented and compared. These statistical analyses are done using ANOVA.

After the basic results are laid out, correlations and regressions are used to determine the relationships between the language tasks—evidentiality and wh-complements—with the Theory of Mind measures.

### 4.1 L1 Turkish versus L1 Bulgarian

**Figure 1:**
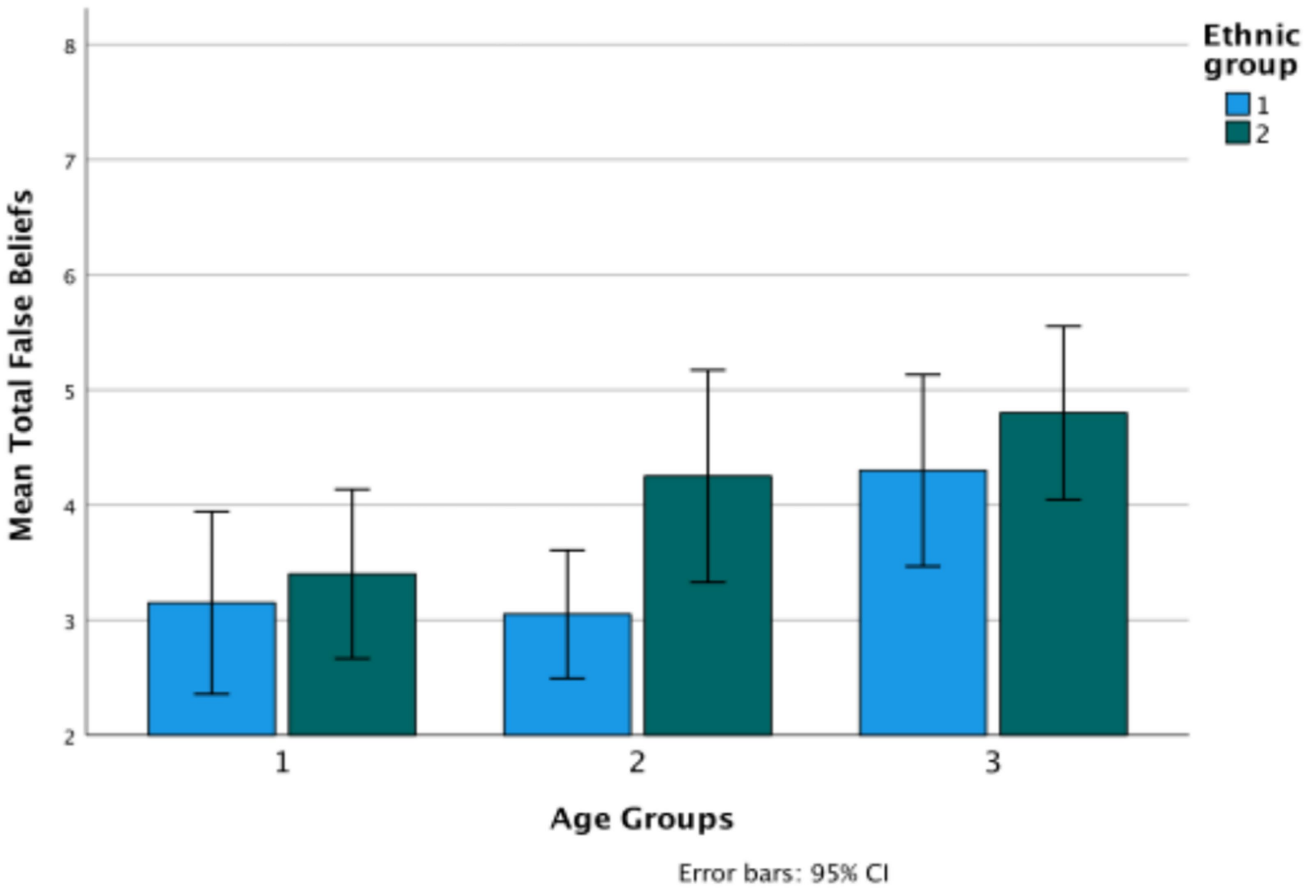
Theory of Mind total in Turkish (Ethnicity 1) versus Bulgarian (Ethnicity 2) ethnic groups.

First, how do the two ethnic groups compare in their L1 on Theory of Mind, understanding evidentials, and the wh-complement task? The following graphs depict the performance of the two groups—Turkish and Bulgarian—in their mother tongue on the three types of tasks. A composite of Theory of Mind is considered first, collapsing together the two classic false belief tasks and the nonverbal task into a single measure, and then testing how the tasks behave separately. Each figure is followed by the results of univariate ANOVAs comparing the ethnic groups and age groups on each measure (Figure 1). Table 2 provides the basic descriptive data.

**Table 2:** Means and standard deviations on each test variable in L1 for each ethnic group.

Ethnicity	Bulgarian mean (S.D.)	Turkish mean (S.D.)
ToM Total	4.15 (1.79)	3.5 (1.65)
Classic ToM	2.33 (1.35)	1.47 (1.08)
Low verbal ToM	1.82 (1.14)	2.03 (0.96)
Wh complements	3.10 (1.97)	3.42 (1.34)
Evidentials	5.12 (2.5)	3.27 (1.9)

**Figure 2:**
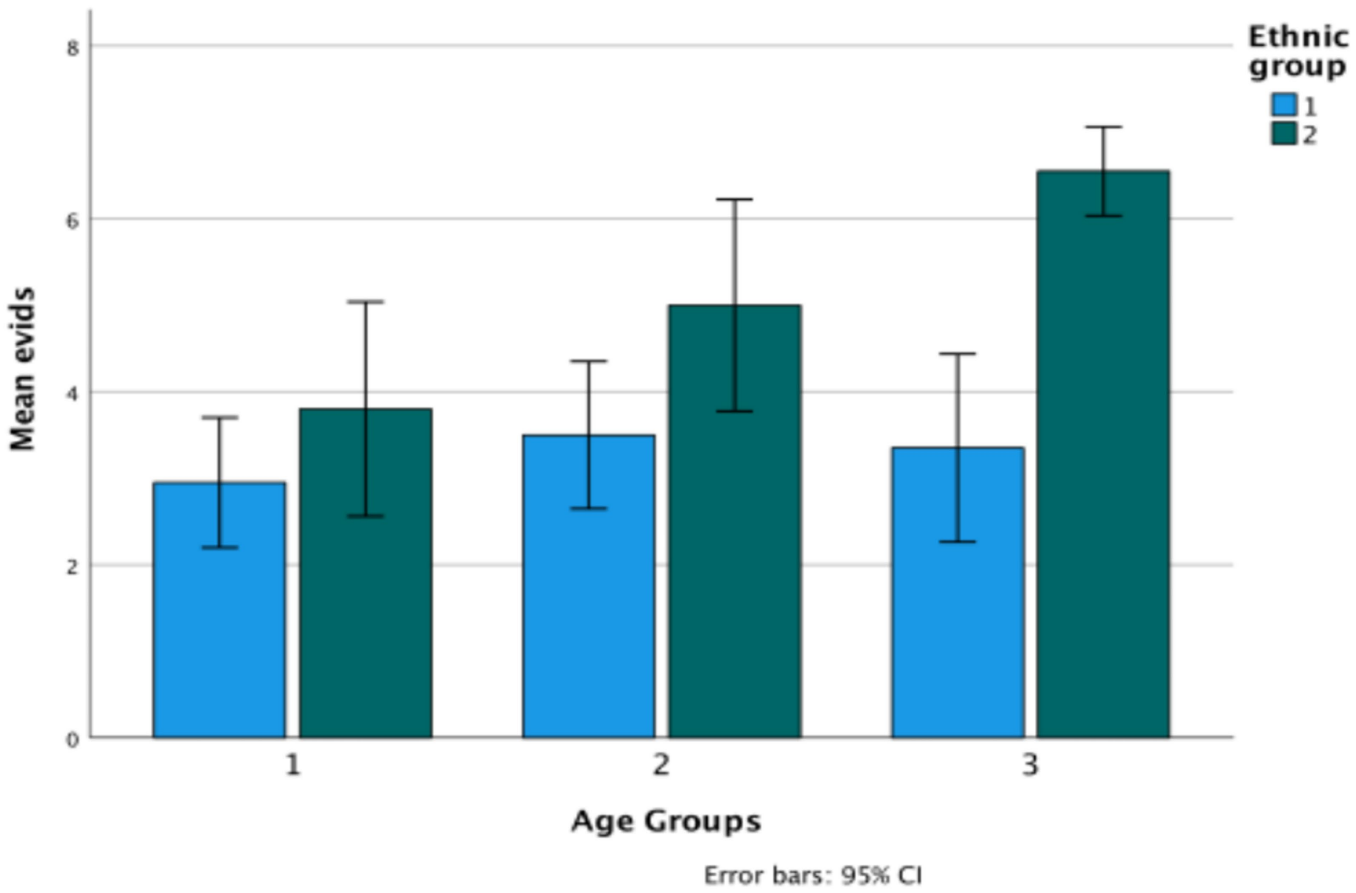
Evidential total score in Turkish (Ethnicity 1) versus Bulgarian (Ethnicity 2) ethnic groups.

The graph shows significant change over age groups (*F*(2,114) = 6.29, *p* = 0.003, *η*^2^ = 0.099), and also a difference between the two ethnic groups (*F*(1,114) = 12.68, *p* = 0.033, *η*^2^ = 0.015) (Turkish *M* = 3.5; Bulgarian *M* = 4.15, difference *p* = 0.033). There is no significant interaction between age and ethnicity. The most significant change over age groups is in the classic tasks (*F*(2,114) = 16.27, *p* < 0.001, *η*^2^ = 0.222), where there is also a difference between the two ethnic groups (*F*(1,114) = 20.04, *p* < 0.001, *η*^2^ = 0.149) (Turkish *M* = 1.47; Bulgarian *M* = 2.33, difference *p* = 0.033). There is also a significant interaction between age and ethnicity, in that there is clearer growth for the Bulgarian children than for the Turkish children on the classic tasks. In contrast, on the low verbal task there are no changes by either age group or ethnicity. Partial correlations controlling for age show low but significant correlations between the classic and the low-verbal tasks only for Turkish (*r* (60) = 0.279, *p* = 0.03) (Figure 2).

**Figure 3:**
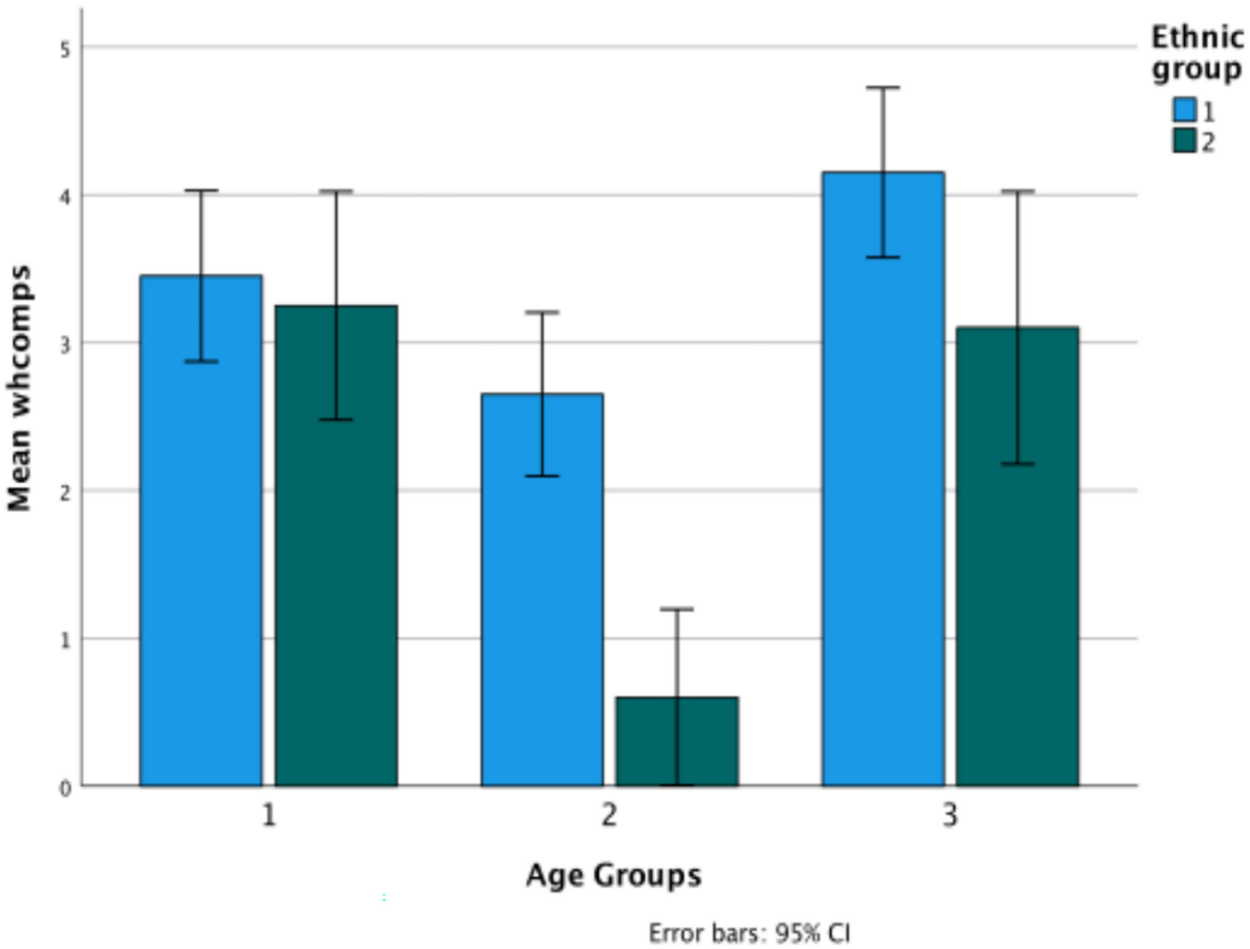
Wh-complement total score in Turkish (Ethnicity 1) versus Bulgarian (Ethnicity 2) ethnic groups.

The graph shows significant change over age groups (*F*(2,114) = 5.67, *p* = 0.004, *η*^2^ = 090), and also a difference between the two ethnic groups (*F*(1,114) = 23.38, *p* < 0.001, *η*^2^ = 170) (Bulgarian *M* = 5.12, Turkish *M* = 3.27, *p* < 0.001). There is also a significant interaction between age and ethnicity (*F*(1.2) = 3.35, *p* < 0.04, *η*^2^ = 0.056), in that there is consistent growth for the Bulgarian children but not for the Turkish children on evidentiality (Figure 3).

In an interesting reversal, the Turkish children do considerably better than the Bulgarian children on the test of wh-complements (*F*(1.114), *p* < 0.001, *η*^2^ = 0.131). There is an interaction with age (*F*(2,114) = 4.07, *p* = 0.02, *η*^2^ = 067), as the middle group of Bulgarian children do very poorly on the task, a fact not explained by their other task performances.

### 4.2 Bulgarian as L1 and L2

The second major descriptive question concerns how the Turkish bilinguals perform in Bulgarian relative to their Bulgarian monolingual peers. The Theory of Mind tasks are split here to test the hypothesis that there may be differences on the verbal but perhaps not on the nonverbal versions of the tasks.

**Figure 4:**
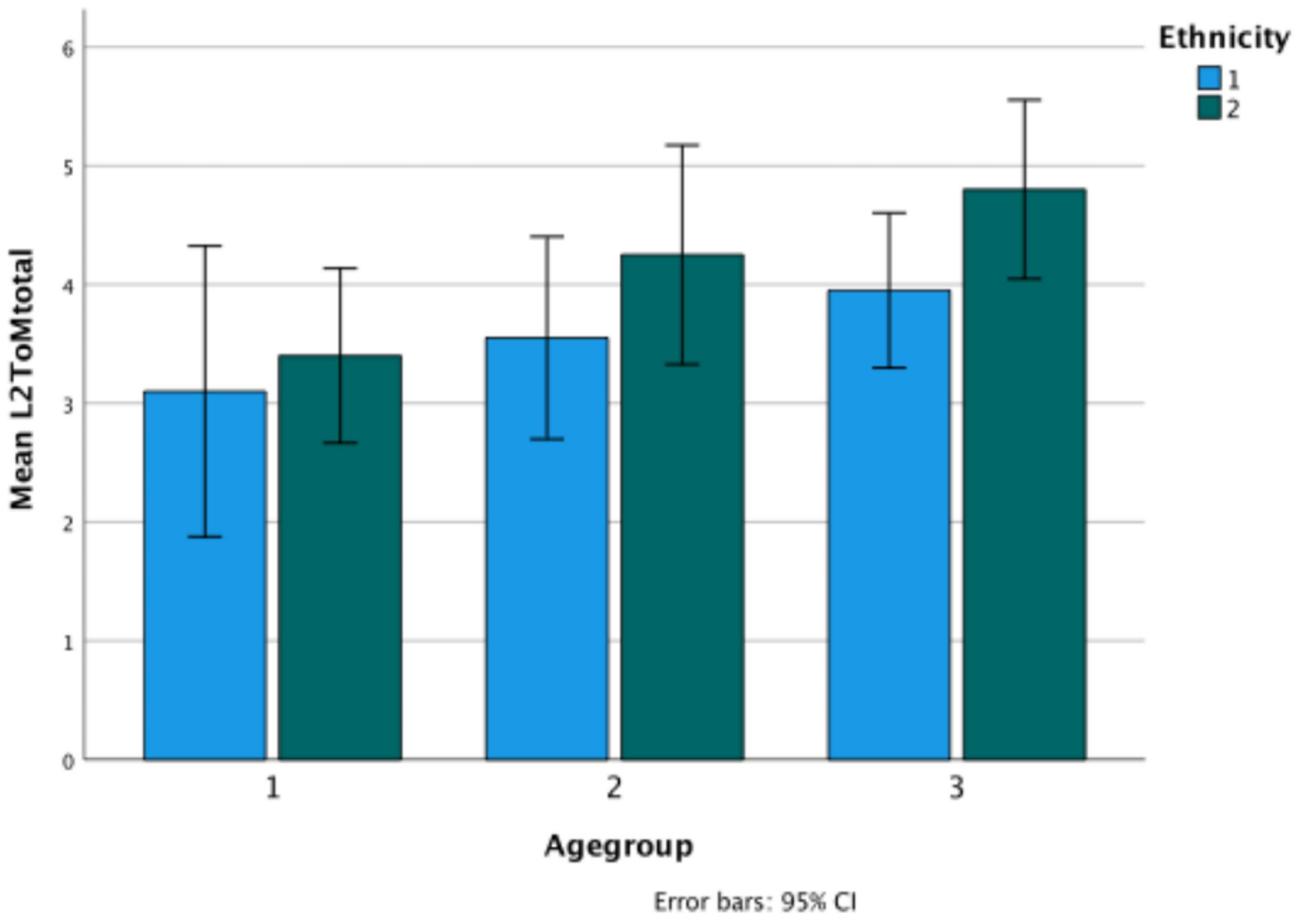
Total Theory of Mind in Turkish (Ethnicity 1) and Bulgarian (Ethnicity 2) ethnic group.

Comparing the various tasks in L1 and L2 Bulgarian, there are several significant differences. First, consider the Theory of Mind measures. Bulgarian children (*M* = 4.2) tested in their L1 slightly outperformed Turkish children (*M* = 3.5) tested in Bulgarian as their L2 (*F*(2,114) = 3.64), though this failed to reach significance overall (*p* = 0.074) (Figure 4).

The classic tasks are contributing all of this effect (*F*(1,114) = 15.51, *p* < 0.001 *η*^2^ = 0.152; Bulgarian *M* = 2.33, Turkish *M* = 1.53, *p* < 0.001), as there is no significant difference on the nonverbal task by ethnicity. Not surprisingly, on both the language tasks, the Bulgarian children outperform the Turkish children on Bulgarian, Wh-complements (*F*(1,114) = 5.8, *p* < 0.02, *η*^2^ = 0.042; Bulgarian *M* = 3.0, Turkish *M* = 2.3, *p* = 0.017); evidentials (*F*(1,114) = 20.5, *p* < 0.001, *η*^2^ = 0.152; Bulgarian *M* = 5.2, Turkish *M* = 3.3, *p* < 0.001).

### 4.3 Comparing L1 and L2 in the Turkish bilingual children

The third descriptive question asks: How do Turkish children compare in their L1 (Turkish) and L2 (Bulgarian)? Here again, the Theory of Mind tasks were divided into the classic verbal versus nonverbal tasks. Table 3 provides basic descriptive data on the two languages of the Turkish bilingual children.

**Table 3:** Means and standard deviations on each test in L1 and L2 in Turkish L1 children.

Language of test	Turkish (L1)	Bulgarian (L2)
Total ToM	3.50 (1.60)	3.53 (1.20)
Classic ToM	1.53 (1.13)	1.47 (1.08)
Low verbal ToM	2.03 (0.96)	2.00 (1.35)
Wh complements	3.42 (1.34)	3.02 (1.52)
Evidentials	3.27 (1.91)	3.30 (2.16)

**Figure 5:**
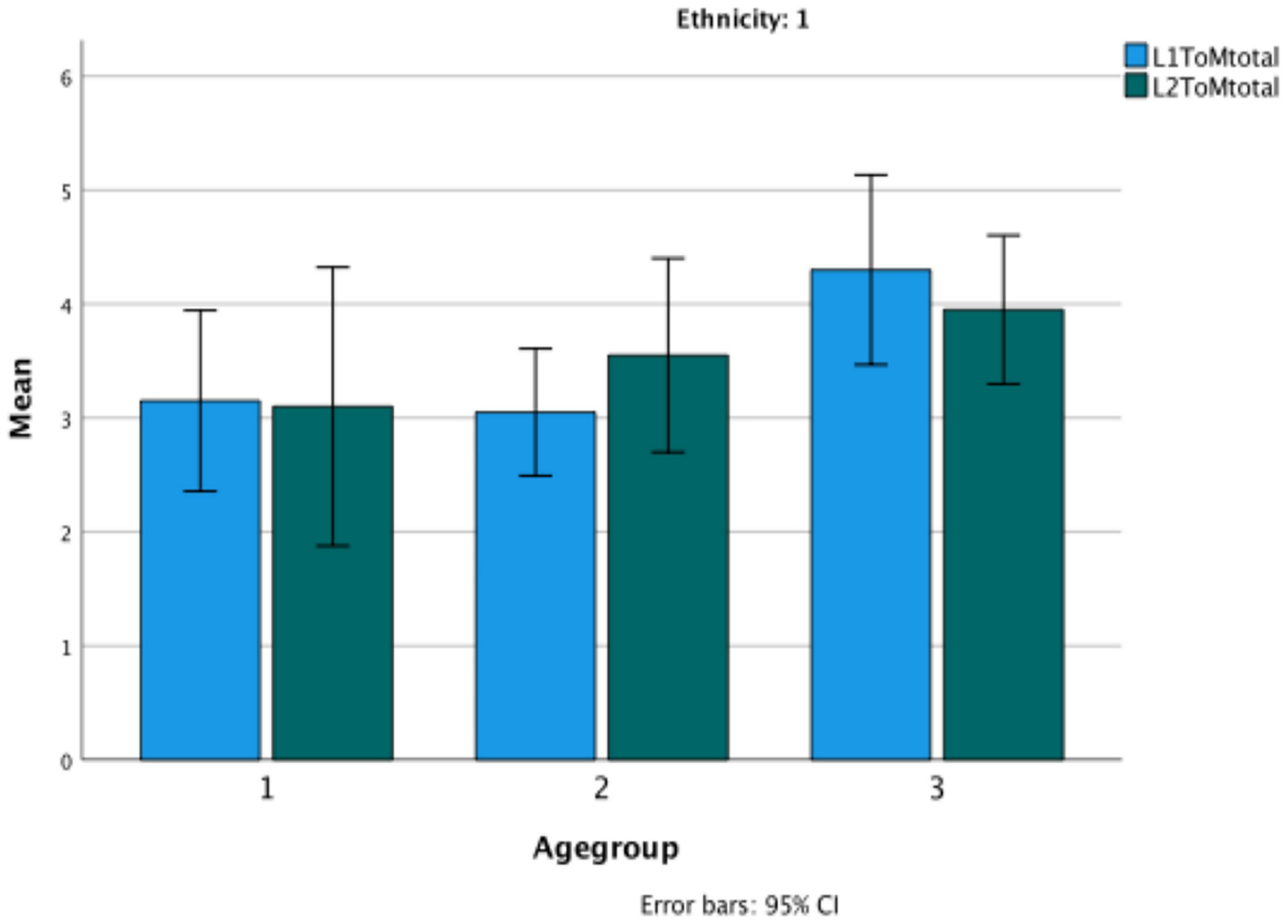
Theory of Mind total in the bilingual children’s L1 versus L2.

**Figure 6:**
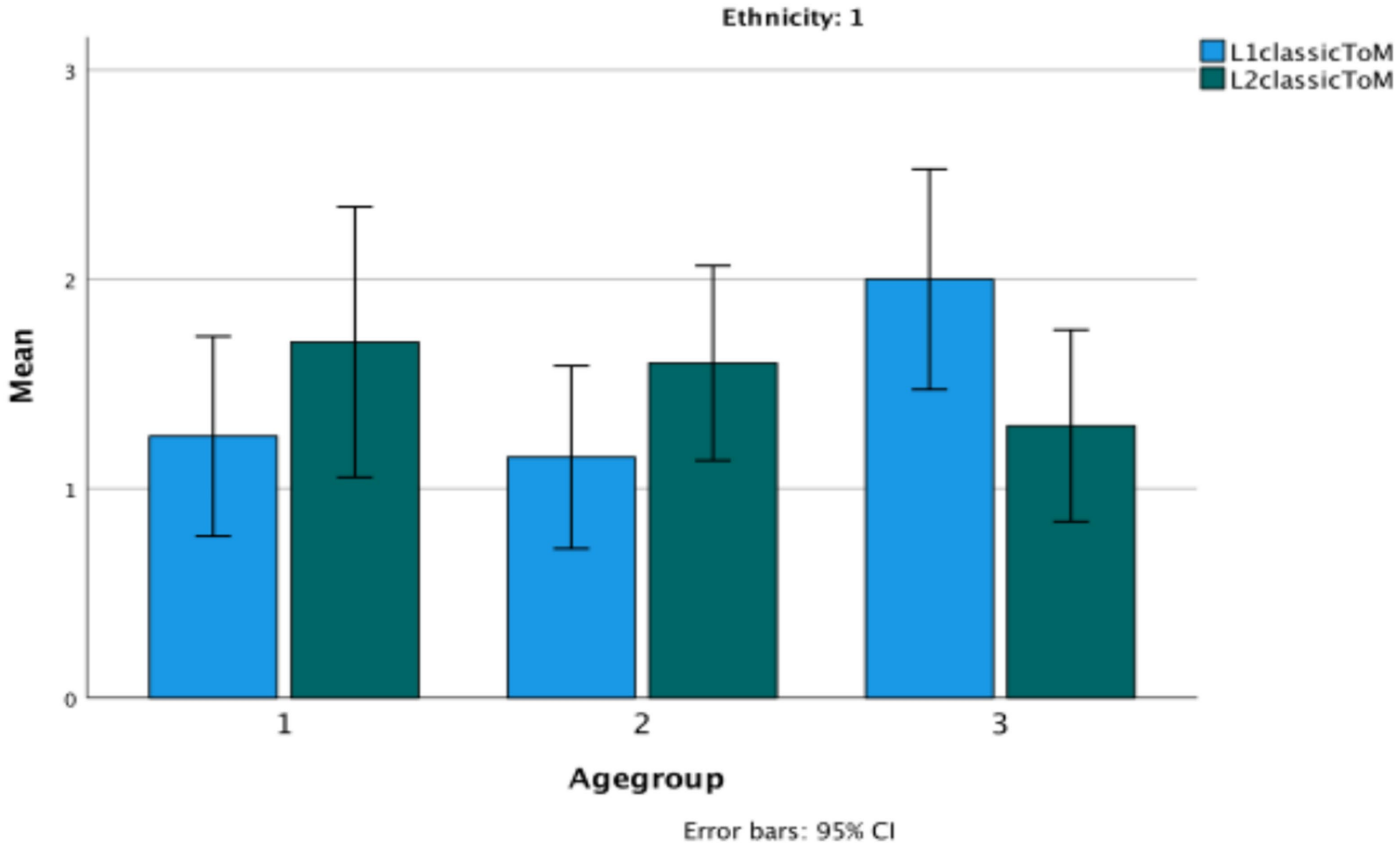
Theory of Mind classic in the bilingual children’s L1 versus L2.

**Figure 7:**
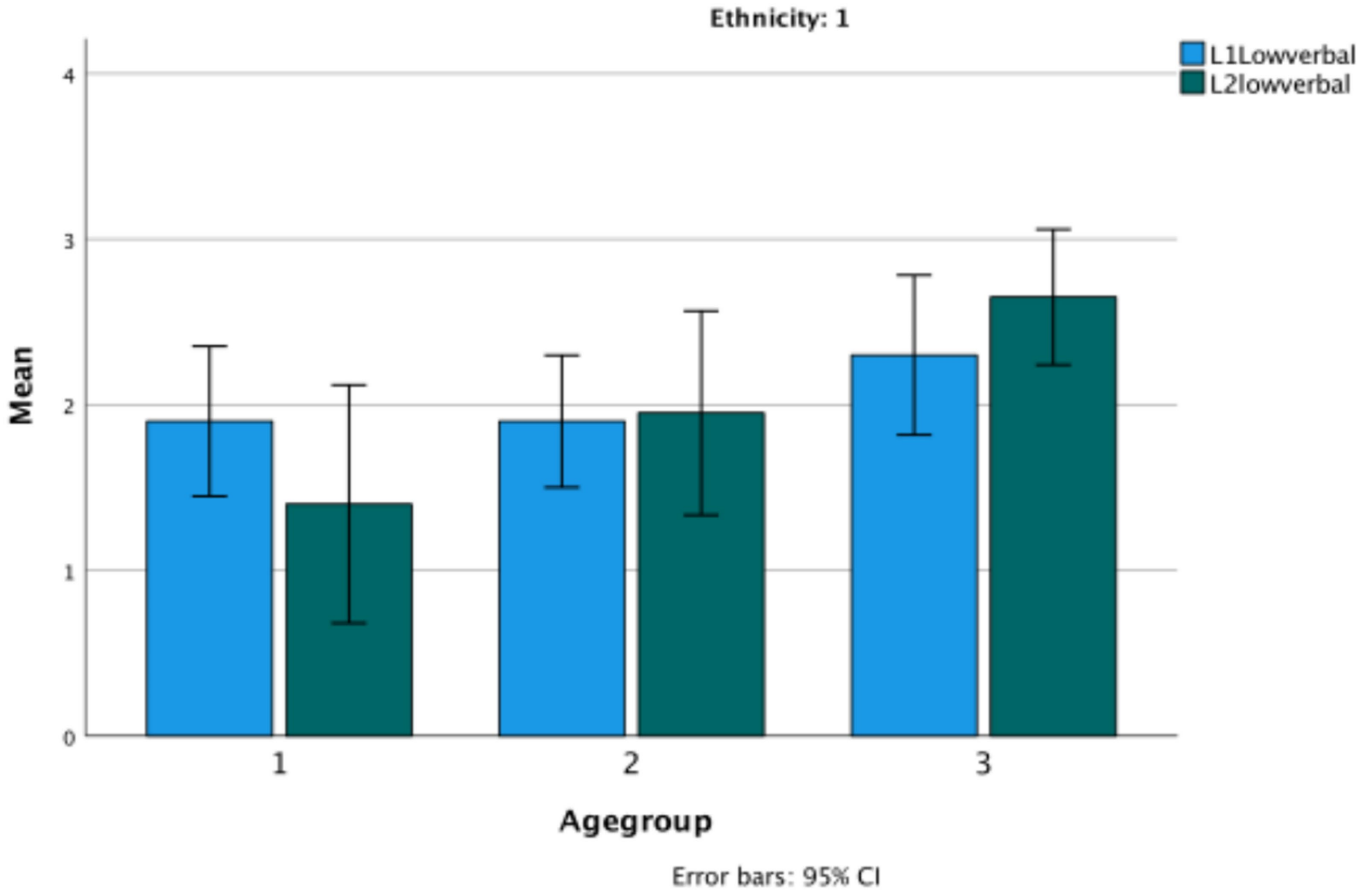
Theory of Mind low verbal in the bilingual children’s L1 versus L2.

**Figure 8:**
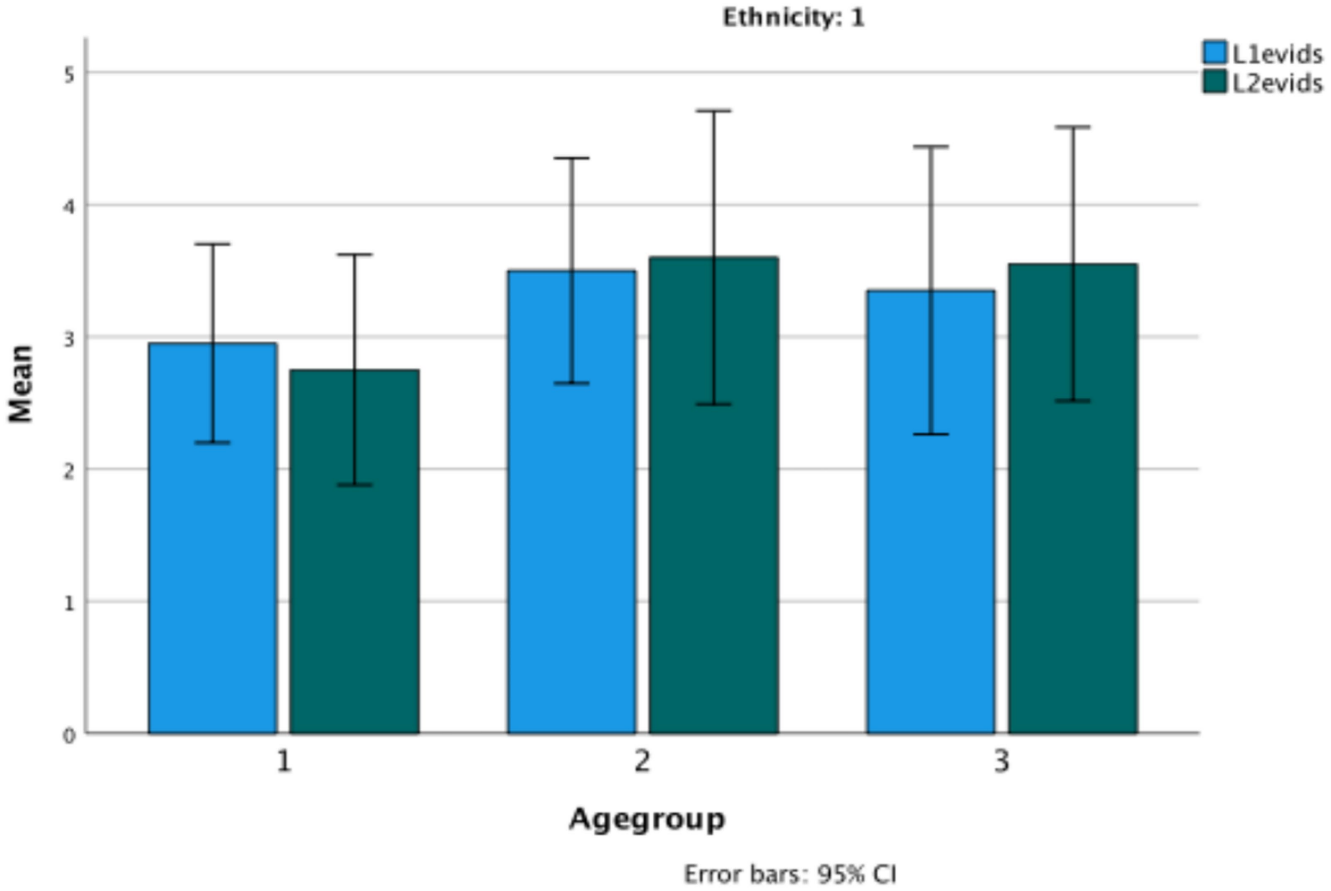
Evidential total score in Bulgarian as L1 versus L2.

**Figure 9:**
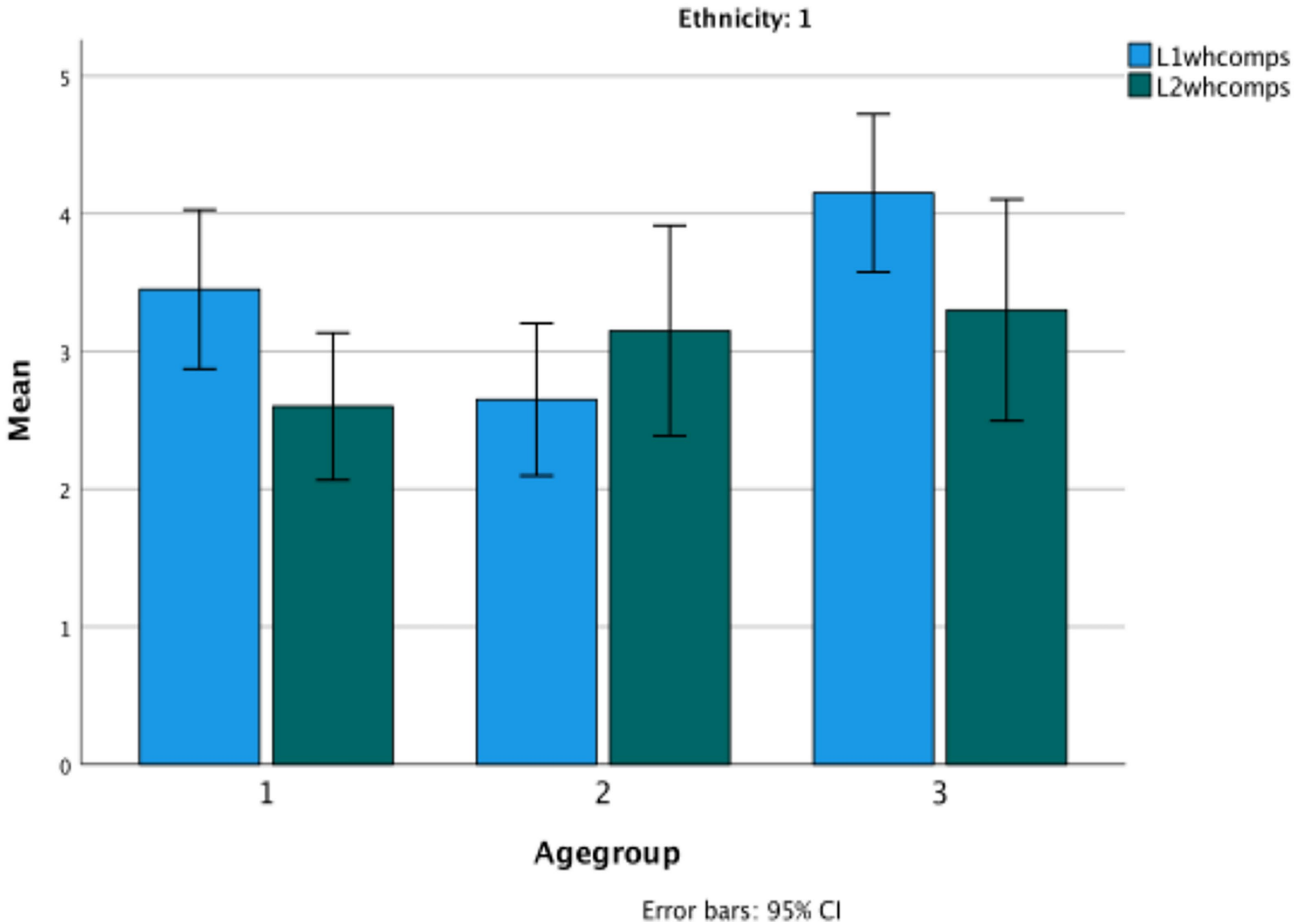
Wh-complement total score in Bulgarian in Turkish bilinguals versus Bulgarian monolinguals.

The statistical analysis used here was a series of repeated measures ANOVAs with age group as a group factor. No significant differences are revealed in the performance of the children in their LI and L2 on any of the Theory of Mind or language measures. In fact, their scores are virtually identical across languages. In only one case is there a significant interaction between the language of the task and age, and that is on classic False Belief tasks (*F*(2,57) = 6.43, *p* < 0.003, *η*^2^ = 0.84). The change is due to the increasingly good performance in Turkish by the third age group (See Figure 5). Again, the low verbal task has a significant but low correlation in each of their languages with the classic FB tasks (Turkish: r (60) = 0.279, *p* = 0.03; Bulgarian: r(60) = 0.300, *p* = 0.02) (Figures 6–9).

### 4.4 Accounting for variance in L1 Theory of Mind scores

In a final set of analyses, linear regressions were used to determine the variables that correlate with success on the false belief tasks. The classic false belief score in the children’s L1 was chosen as the dependent variable. As predictors, age group was entered, followed by nonverbal false belief, wh-complements, and evidentials. The regression (Table 4) reveals that when the groups are considered together, performance on evidentials contributes significantly to the variance on classic false belief tasks, in addition to age.

**Table 4:** Regression model summary for L1 for all participants.

Model	*R*	*R* ^2^	Adj. *R*^2^	Beta	*t* value	*p* value	*R*^2^ change	*F* value	*p* value
Age group	0.428	0.183	0.176	0.428	5.15	0.001	0.183	26.493	<0.001
Age group + Ethnicity	0.545	0.297	0.285	0.428 0.337	5.23 4.34	0.001 0.001	0.113	18.856	<0.001
Age group, + Ethnicity, + L1 wh comps	0.560	0.314	0.296	0.437 0.295 −1.37	5.66 3.65 −1.75	0.00 0.001 0.092	0.017	2.878	0.092
Age group, + Ethnicity, + L1 wh comps, L1 evids	0.592	0.350	0.327	0.378 0.212 −0.133 0.216	4.8 2.48 1.69 2.53	0.001 0.015 0.095 0.013	0.036	6.419	0.013

Since ethnicity has a significant effect, a second analysis reran the regressions with the two groups separately (Table 5).

**Table 5:** Regression model summary for L1 for Bulgarian.

Model	*R*	*R*	*R* ^2^	Adj. *R*^2^	Beta	*t* value	*p* value	*R*^2^ change	*F* value	*p* value
Age group		0.595	0.354	0.343	0.595	5.64	0.001	0.354	31.821	<0.001
Age group L1 wh comps		0.628	0.394	0.373	0.589 −0.200	5.71 −1.94	0.001 0.057	0.040	3.762	0.057
Age group L1 wh comps L1 evids		0.675	0.455	0.426	0.461 −0.216 0.279	4.16 −2.18 2.51	0.001 0.033 0.015	0.061	6.288	0.015

In the Bulgarian children, their L1 classic Theory of Mind is affected by age (*R*^2^ change = 0.354, *F*(1,55) = 31.8, *p* < 0.001), and significantly by their understanding of evidentials (*R*^2^ change = 0.061, *F*(1,55) = 6.29, *p* = 0.015). Table 5 provides the details.

In contrast, in the Turkish children, command of L1 classic false belief is a product only of age (*R*^2^ change = 0.082, *F*(1,55) = 5.1, *p* = 0.027), with the language measures failing to contribute at all.

In a final regression, the bilinguals’ L2 classic false belief task was used as the dependent variable. Is there transfer from acquiring L1 false belief, either verbal or low-verbal, or from the language variables in either language? Predictor variables were age, classic false belief in L1, wh-in L1 and L2, and evidentials in L1 and L2. The only significant contributing factor to L2 classic false belief was L1 classic false belief (*R*^2^ change = 0.154, *F*(1,57) = 10.7, *p* = 0.002).

## 5 Discussion

First, a summary of the complex findings is in order. Bulgarian monolinguals outperform Turkish bilinguals on classic Theory of Mind tasks, but not on a nonverbal Theory of Mind task. In this age range 3;6 to 5, not many children are showing a high level of success. The two groups differ on the language tasks in their respective L1s, but in different directions: Bulgarian children do better on evidentials in their L1, and Turkish children do better on wh-complements in their L1.

In Bulgarian, it is not surprising that Bulgarian children tested in their L1 outperform the Turkish children for whom Bulgarian is their L2, on all the tasks except for the nonverbal Theory of Mind task where the groups are equivalent. In testing performance in their two languages, the Turkish bilinguals perform equivalently well on all tasks, suggesting they already have sufficient command of the language required in both Turkish and Bulgarian. The oldest group are slightly better in Turkish than Bulgarian on the classic false belief tasks.

The regressions suggest a close relationship in Bulgarian children tested in L1 between their performance on classic false belief tasks and their command of language, more so with evidentials than wh-complements. Low verbal tasks are not predictive. However, the language tasks do not predict either L1 or L2 false belief in Turkish children. In contrast, the best predictor for Turkish-speaking children is their performance on the nonverbal Theory of Mind test.

The performance of the Turkish bilinguals is much less a product of age than for the monolingual Bulgarians. This suggests greater variability in the group, perhaps caused by differential competence in the two languages, or SES differences making for greater variability. There is a lot of variance that cannot be accounted for in the Turkish Theory of Mind performance. The low verbal Theory of Mind task did equalize performance across the mono- and bi-lingual groups, and it correlated with the classic false belief tasks. It is helping the Turkish children in their classic FB performance in L1 and L2.

Given previous research reviews suggesting the superiority of bilingual children on ToM tasks relative to monolingual children, the results are unexpected. The bilingual children did not perform as well as the monolingual children when tested in Bulgarian (their L2), nor did they test as well as the Bulgarian monolinguals when tested in their L1. Two factors come in to explain the discrepancy. First, these children were quite young by the standards of existing research and seem to be still developing the necessary skills in both ToM and language. Second, the children are from quite different social classes. The Bulgarian children came from middle class SES households, with the Turkish bilinguals coming from low SES communities. Though SES has not always shown a significant effect before (Nguyen and Astington, 2014), the SES discrepancy might be greater in this study.

Interestingly, the difference between the groups was marked for the verbal ToM tasks but not for the low verbal ToM tasks, where the two groups were equivalent. This suggests that still-developing language skills may be holding back the bilingual children from success on the verbal ToM tasks in both languages, in which they perform equivalently. At 3, the Turkish bilingual children are still too young to have developed the complex linguistic competence and vocabulary in their L1 Turkish, and that is the age that they begin learning Bulgarian. The regressions confirm that the Bulgarian children succeed on classic false belief tasks contingent on their command of language, and more so with understanding evidentials than wh-complements. In contrast, the Turkish bilinguals’ language skills on evidentials and wh-complements do not predict either L1 or L2 false belief. The only predictor for the bilingual children is their performance on the nonverbal Theory of Mind test. It may be that the successive bilingualism of these children, even though it happens early in development, will not pay off until an older age. Kyuchukov (2023) found that wh-complements predicted FB in older Turkish-German bilingual children. For children in the present study, their nonverbal skills in understanding false belief contribute to their skills on the verbal tasks. The monolingual Bulgarian children seem to benefit from their additional linguistic skills on the verbal FB tasks.

The research suggests that there is greater nuance to the story of the development of Theory of Mind in different communities of bilinguals. Some recent work suggests that a binary approach to categorizing children as bilinguals may be a mistake (Marian and Hayakawa, 2021). It may be too quick to conclude that SES is not relevant to the development of skills in ToM for bilingual children, especially in cases where one language is very different status or not given sufficient support in educational settings. The timing of exposure to a second language may matter if the child is not simultaneously learning both languages: is one language sufficiently developed to support complex reasoning skills before the second exposure begins?

## 6 Conclusion

This research was conducted to test the performance level of young Turkish-Bulgarian bilinguals on ToM tasks, and the role their language skills played in ToM development. Two language tests were selected, one on wh-complements and one on evidentiality, both of which have been previously linked to ToM development.

With respect to the linguistic prediction of FB reasoning: comprehension of evidentiality contrasts comes out as a more important predictor here than understanding wh-complements, which is a new finding, and only in Bulgarian L1. The determinants of success in Turkish bilinguals are unclear in this group and need further exploration, possibly with an older age group of 5- to 7-year olds in which the language skills and classical ToM skills are more advanced.

## Data Availability

The original contributions presented in the study are included in the article/supplementary material, further inquiries can be directed to the corresponding author.

## Footnotes

^1^The tests were designed in cooperation with Jill de Villiers, and the illustrations were done by her. In the study here they are used with her permission.
